# Molecular and Structural Insights of *carO* Gene Variations in Carbapenem-Resistant *Acinetobacter baumannii*

**DOI:** 10.3390/antibiotics15090828

**Published:** 2026-08-26

**Authors:** Ploychomphoo Poollak, Nattita Srichomthong, Kittaporn Yartirat, Phuchit Pooruk, Rachanon Kiewdee, Nitchawat Paiyabhroma, Sattaporn Weawsiangsang, Sittichai Urtgam, Nontaporn Rattanachak, Touchkanin Jongjitvimol, Jirapas Jongjitwimol

**Affiliations:** 1 Master of Science Program in Medical Technology, Department of Medical Technology, Faculty of Allied Health Sciences, Naresuan University, Phitsanulok 65000, Thailand; ploychomphoop67@nu.ac.th; 2 Biomedical Sciences Program, Faculty of Allied Health Sciences, Naresuan University, Phitsanulok 65000, Thailand; nattitas68@nu.ac.th; 3 Department of Medical Technology, Faculty of Allied Health Sciences, Naresuan University, Phitsanulok 65000, Thailand; kittaporny66@nu.ac.th (K.Y.); phuchitp66@nu.ac.th (P.P.); rachanonk66@nu.ac.th (R.K.); nitchawatp@nu.ac.th (N.P.); 4 Integrative Biomedical Research Unit (IBRU), Faculty of Allied Health Sciences, Naresuan University, Phitsanulok 65000, Thailand; 5 Department of Optometry, Faculty of Allied Health Sciences, Naresuan University, Phitsanulok 65000, Thailand; sattapornw@nu.ac.th; 6 Biology Program, Faculty of Science and Technology, Pibulsongkram Rajabhat University, Phitsanulok 65000, Thailand; sittichai.u@psru.ac.th (S.U.); nontaporn.r@psru.ac.th (N.R.); touchkanin@psru.ac.th (T.J.); 7 Centre of Excellence in Biomaterials, Faculty of Science, Naresuan University, Phitsanulok 65000, Thailand

**Keywords:** *Acinetobacter baumannii*, carbapenem resistance, CarO, outer membrane protein, molecular epidemiology, antimicrobial resistance, protein structure prediction

## Abstract

**Background/Objectives:** Carbapenem-resistant *Acinetobacter baumannii* (CRAB) is a WHO critical-priority pathogen, and variations in the outer membrane porin CarO are one mechanism implicated in carbapenem resistance. No prior study has characterized *carO* mutation patterns among *A. baumannii* in Tak, Thailand. This study aimed to characterize *carO* mutation patterns in clinical isolates, evaluate their association with CRAB, reconstruct phylogeny, and predict the structural consequences of CarO representatives relative to a crystallographic reference structure (PDB 4RL9). Seventy-seven *A. baumannii* isolates were recovered from Mae Sot Hospital, and re-confirmed by PCR based on the presence of *bla*_OXA-51_-like gene. **Results:** The *carO* gene was sequenced and mutations compared between CRAB and carbapenem-susceptible (CSAB) isolates. Of the 77 isolates, 70 yielded successfully *carO* sequence data by next-generation sequencing (NGS), including 64 CRAB (91.43%) and 6 CSAB isolates. Two overlapping mutation clusters were significantly associated among CRAB isolates (73.44% and 75.00%; *p* = 0.0008 and *p* = 0.0006) and were absent from all 6 CSAB isolates (8.57%). Phylogenetic analysis resolved 3 clinical lineages, with variant III predominating. AlphaFold2 models predicted structural divergence among CarO variants with the single variant IV isolate showing the most extensive predicted remodeling of the CarO barrel. **Conclusions:** *carO* mutation was strongly associated with carbapenem resistance in this Thai cohort. However, these findings do not establish a direct causal role of *carO* mutation in carbapenem resistance. Predicted structures were consistent with potential alterations of porin function. These findings provide a regional characterization of *carO*-mediated resistance and support further functional validation of CarO as a resistance marker.

## 1. Introduction

Over the past several decades, *Acinetobacter baumannii* has emerged as a major nosocomial pathogen, causing substantial morbidity and mortality associated with antimicrobial resistance worldwide [1,2]. It is formally classified among the ESKAPE organisms, which were recognized for combining high pathogenicity with a persistent capacity to evade antimicrobial therapy [3]. Its genetic adaptability allows it to persist in hospital environments for extended periods, contributing to its role as a major source of healthcare-associated infections [4,5]. Clinically, *A. baumannii* is associated with a wide range of infections, including ventilator-associated pneumonia, bloodstream infections, urinary tract infections, meningitis, and surgical site infections [5,6]. It frequently exhibits multidrug-resistant (MDR) or extensively drug-resistant (XDR) phenotypes, including resistance to carbapenems, such as imipenem, meropenem, and doripenem [5].

Carbapenems, a class of β-lactam antibiotics, have traditionally served as last-resort agents for severe infections caused by MDR *A. baumannii* once other β-lactams have failed [7]. Since carbapenem-resistant *A. baumannii* (CRAB) was first documented in 1985, CRAB strains have now disseminated globally [7]. Accumulating evidence from a systematic review demonstrates that patients with CRAB infections experience significantly higher mortality rates than those with CSAB infections [8]. The severity of this problem led the World Health Organization to designate CRAB as a critical-priority pathogen in 2017 and to retain it as a critical-priority pathogen in 2024 [9]. This highlights the continued need for innovation in diagnostics, therapeutics, and antimicrobial resistance control [9].

Mechanistically, carbapenem resistance in *A. baumannii* arises through several mechanisms [10,11]. For example, these mechanisms include enzymatic drug inactivation by OXA-type carbapenemases and metallo-β-lactamases, efflux pump overexpression (e.g., RND family), alteration of penicillin-binding protein targets, and reduced outer membrane permeability through porin loss or modification [10,11]. Among the porins implicated in the last of these mechanisms, CarO (Carbapenem-associated resistance protein) has attracted particular attention. Structural work has shown that CarO forms an eight-stranded β-barrel channel selective for small basic amino acids such as ornithine and glycine as well as carbapenems [12,13]. Genomic diversity studies have further demonstrated that *carO* is not a uniform locus [14]. The *carO* gene exhibits multiple sequence variants circulating among clinical *A. baumannii* populations, and this diversity may be influenced by horizontal gene transfer and recombination [14]. Several functional studies have supported the clinical relevance of this diversity [15,16,17]; for instance, Zhu et al. found that *carO* mutation, evaluated alongside three-dimensional protein modeling, distinguished imipenem-resistant from imipenem-susceptible isolates [15]. Moreover, Lee et al. described an insertion sequence, ISAba10, that disrupted *carO* and reduced carbapenem permeability during a South Korean hospital outbreak [16], and Labrador-Herrera et al. linked intact and mutated *carO* genotypes to differences in both antimicrobial susceptibility and virulence among bloodstream isolates from ICU patients [17]. A large Egyptian resistome survey similarly found an association between *carO* mutation and carbapenem resistance [18].

Despite this accumulating international evidence, molecular characterization of *carO*-mediated carbapenem resistance remains comparatively sparse within Thailand, where national surveillance data document a rising prevalence of *A. calcoaceticus-baumannii* complex infection (from approximately 8% in 2000 to 14% in 2022), with resistance rates in some settings now exceeding 70% and disproportionately concentrated in intensive care units [19]. However, the distribution of *carO* sequence variation and its relationship with carbapenem resistance among clinical *A. baumannii* isolates in northern Thailand remain poorly characterized. This gap is particularly evident at the regional level, as no prior study has characterized *carO* variation among clinical *A. baumannii* isolates from Tak Province, a region where CRAB accounted for 68.3% of *A. baumannii* infections at Mae Sot Hospital in 2024. This study, therefore, aimed to (1) characterize the sequence variation in the *carO* gene, including translated amino acid changes, in *A. baumannii* isolates recovered from clinical specimens at Mae Sot Hospital, Tak Province, Thailand, (2) evaluate whether specific *carO* sequence variants are statistically associated with CRAB, (3) reconstruct the phylogenetic tree of the clinical isolates compared to the global reference strains, and (4) predict the structural consequences of representative CarO variants compared with the available crystallographic structure of a reference CarO protein.

## 2. Results

### 2.1. General Characteristics of Clinical Isolates at the First Identification as A. calcoaceticus-baumannii Complex

In the present study, a total of 83 *A. calcoaceticus-baumannii* complex isolates were initially recovered from routine clinical specimens at the Clinical Microbiology Laboratory of Mae Sot Hospital, Tak, Thailand. The distribution of these specimens comprised sputum (*n* = 54; 65.06%), pus (*n* = 17; 20.48%), blood (*n* = 4; 4.82%), body fluids (*n* = 4; 4.82%), and urine (*n* = 4; 4.82%), as detailed in Table 1. The predominant proportion of the samples was obtained from Thai patients (*n* = 52; 62.65%), whereas the remaining specimens were obtained from Burmese patients (*n* = 31; 37.35%). Furthermore, approximately two-thirds of the clinical specimens were obtained from male patients (*n* = 55; 66.27%), with the overall study population exhibiting an average age of 57.51 years.

### 2.2. Genotypically Confirmed A. baumannii (bla_OXA-51_-like Positive) Were Included in the Study

We confirmed the presence of *bla*_OXA-51_-like gene as a species-specific marker for *A. baumannii* using PCR. Amplicons of the *bla*_OXA-51_-like gene of *A. baumannii* were detected in 77 out of 83 isolates. The six *bla*_OXA-51_-like negative strains were excluded from the population. Bacterial antimicrobial susceptibility testing (AST) was performed using the automated VITEK^®^ 2 COMPACT system with AST-N288 cards. Among the *A. baumannii* isolates, 70 isolates (90.91%) were carbapenem-resistant *A. baumannii* (CRAB), whereas 7 isolates (9.09%) were carbapenem-susceptible *A. baumannii* (CSAB) strains. Based on their resistance profiles, the isolates were classified as extensively drug-resistant (XDR; *n* = 69, 89.61%), multidrug-resistant (MDR; *n* = 1, 1.30%), and susceptible (*n* = 7, 9.09%). The clinical characteristics of the population stratified by carbapenem resistance status are summarized in Table 2. No statistically significant differences were observed between the CRAB and CSAB groups across any of the evaluated parameters, indicating comparable baseline characteristics between the two groups.

According to the demographic distribution, Thai nationals comprised the majority of the overall population (63.64%, *n* = 49), representing 65.71% (*n* = 46) of the CRAB group and 42.85% (*n* = 3) of the CSAB group. Burmese patients accounted for 34.29% (*n* = 24) and 57.14% (*n* = 4) of the CRAB and CSAB groups, respectively. This variation in nationality between the two groups did not reach statistical significance (*p* = 0.2488). In terms of gender, a distinct male predominance was observed in the total population (70.13%, *n* = 54), which was consistently reflected within both the CRAB (71.43%, *n* = 50) and CSAB (57.15%, *n* = 4) groups, demonstrating no statistically significant differences (*p* = 0.4203). The isolates were predominantly recovered from internal medicine wards (42.86%, *n* = 33) and the intensive care unit (ICU) (36.36%, *n* = 28), followed by surgical wards (14.29%, *n* = 11). When performing a binary comparison of distributions between internal medicine wards and all other combined hospital units, no significant difference was identified between the CRAB and CSAB groups (*p* > 0.4539). Sputum was identified as the primary specimen source, accounting for 66.23% (*n* = 51) of all clinical isolates, with remarkably uniform proportions observed in both the CRAB (65.71%, *n* = 46) and CSAB (71.43%, *n* = 5) groups. Pus was the second most frequent specimen type overall (20.78%, *n* = 16), comprising 20.00% (*n* = 14) of CRAB and 28.57% (*n* = 2) of CSAB strains. A comparative statistical analysis evaluating sputum versus all non-sputum specimen types combined revealed no statistically significant differences between the two groups (*p* = 1.0000).

### 2.3. Antimicrobial Susceptibility Profiles and Phenotypic Distribution

**Figure 1:**
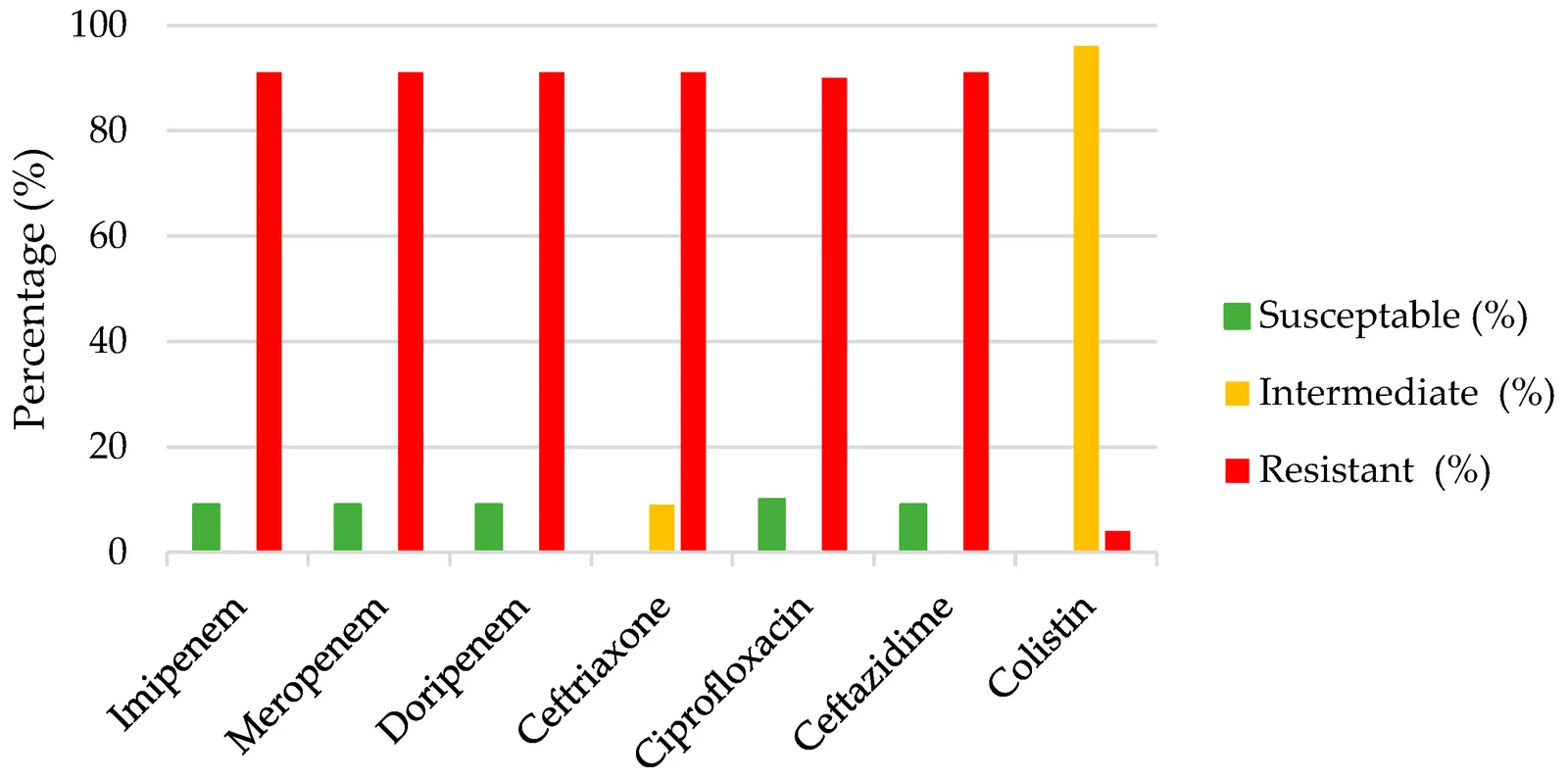
Antimicrobial susceptibility profile of clinical *A. baumannii* isolates. Percentage distribution of susceptibility categories (susceptible, intermediate, and resistant) among clinical *A. baumannii* isolates against seven antimicrobial agents, determined by standard antimicrobial susceptibility testing and interpreted according to CLSI.

A total of 77 clinical *Acinetobacter baumannii* isolates were evaluated for antimicrobial susceptibility using the VITEK^®^ 2 Compact system. The overall susceptibility, intermediate, and resistance profiles across the tested antibiotics are illustrated in Figure 1. Phenotypic analysis revealed a dominance of extensively drug-resistant (XDR) and multidrug-resistant (MDR) profiles, accounting for 89.61% (*n* = 69) and 1.30% (*n* = 1) of the isolates, respectively, while only 9.09% (*n* = 7) exhibited non-resistant phenotypes. The clinical isolates exhibited broad-spectrum resistance across multiple antibiotic classes, most notably against β-lactams and fluoroquinolones. Specifically, high resistance rates were observed for carbapenems, with 90.91% (*n* = 70) of isolates displaying resistance to imipenem, meropenem, and doripenem (defining the CRAB cohort). Cephalosporins and fluoroquinolones similarly showed severe resistance profiles. Notably, ceftriaxone demonstrated 100% non-susceptibility (*n* = 77/77, 0% susceptible) across both CRAB and CSAB strains, while ceftazidime and ciprofloxacin exhibited resistance rates surrounding 90%. Overall, these findings highlight the high prevalence of MDR and XDR phenotypes and the limited antimicrobial treatment options available for these isolates.

### 2.4. Comparative Sequence Analysis of CarO Reveals a Strong Association Between Distinct Polymorphic Patterns and Carbapenem Resistance

**Figure 2:**
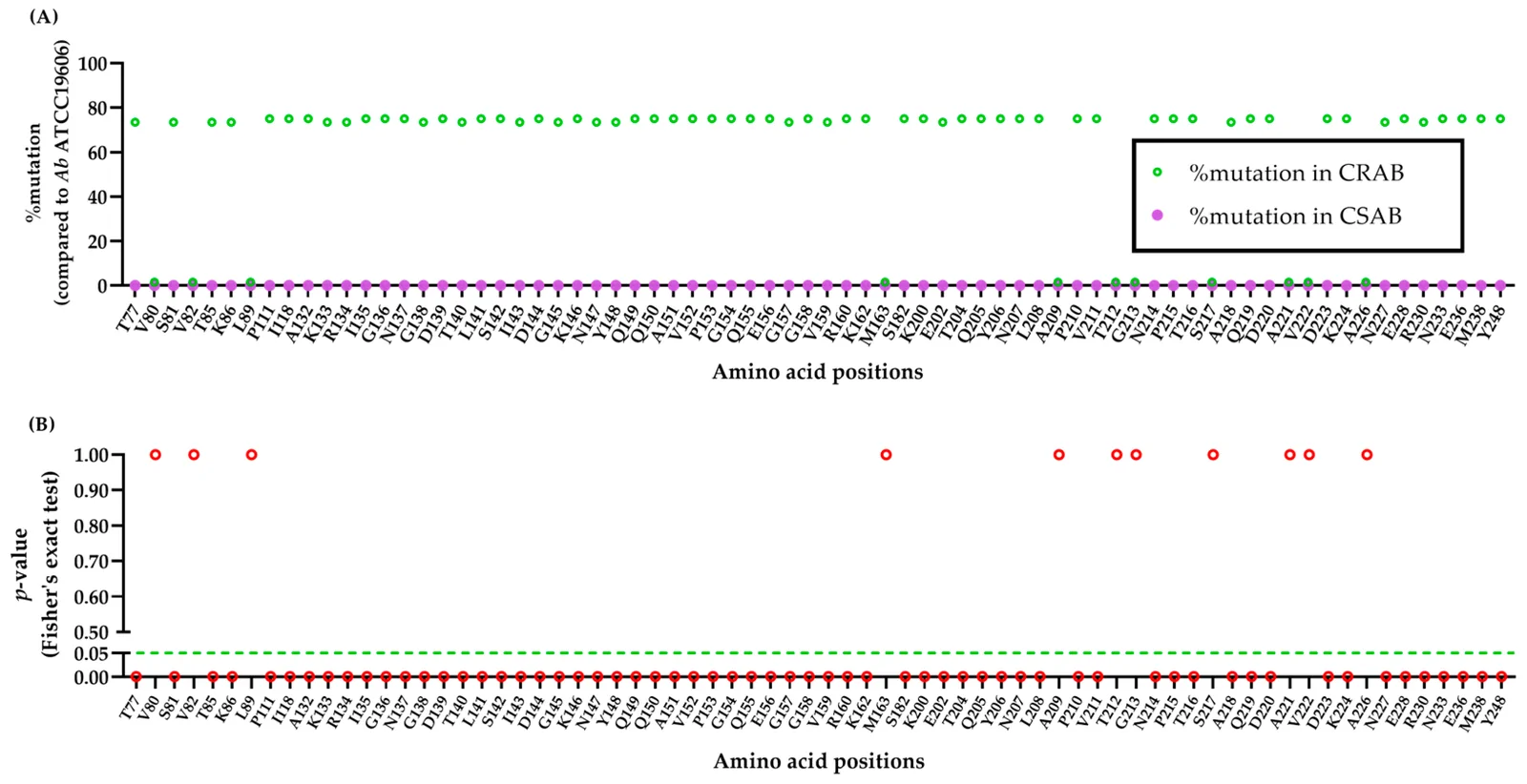
The distribution of mutations occurring in the clinical CRAB (green circle) and CSAB (purple circle) isolates (**A**) compared to the *A. baumannii* ATCC19606 reference strain, and *p*-values from Fisher’s exact test (red circle) show the statistical significance level (green dotted line at a *p*-value of 0.05) of these amino acid mutations (**B**).

Comparative mutational analysis of the CarO amino acid sequence relative to the *A. baumannii* ATCC19606 reference strain revealed distinct polymorphic patterns between carbapenem-resistant *A. baumannii* (CRAB, *n* = 64) and carbapenem-susceptible *A. baumannii* (CSAB, *n* = 6) isolates. During the initial PCR step, 70 isolates amplified robustly without any failure, whereas the remaining seven isolates (comprising 6 CRAB and 1 CSAB) yielded only faint or weak bands. Although these seven samples were subjected to NGS to recover sequence data, they failed to yield complete sequences, showing insufficient coverage across the *carO* locus, which suggests possible *carO* gene disruption, deletion, or PCR product insufficiency. Among 70 isolates, the amino acid variations were statistically associated with CRAB isolates (*p* = 0.0002). The distribution and statistical significance of individual amino acid changes are shown in Figure 2.

A prominent cluster of amino acid variations was exclusively identified within the CRAB cohort, demonstrating a statistically significant difference compared to the CSAB group (*p* < 0.05). Specifically, these variations were observed in either 75.00% or 73.44% of the CRAB isolates (*n* = 48/64, *p* = 0.0006; *n* = 47/64, *p* = 0.0008) and were distributed across multiple amino acid positions. Remarkably, none of these specific variations were detected in any of the susceptible CSAB strains (*n* = 0, 0%). False discovery rate adjustment was further applied using the Benjamini and Hochberg method to calculate *q*-values (Table S1), confirming that there was a true difference in amino acid mutations in CRAB compared to those in the CSAB group.

Conversely, several single-nucleotide polymorphisms appeared sporadically and failed to establish statistical relevance (*p* > 0.9999). These low-frequency baseline variations were restricted to solitary occurrences (*n* = 1) within the CRAB arm at positions V80L, V82I, L89V, M163L, A209F, T212A, G213A, S217N, A221D, V222L, and A226E (in which no mutations were observed in either group), as shown specifically in Table S1.

### 2.5. Clinical A. baumannii Isolates Cluster into Three Lineages, with Variant III Predominating

**Figure 3:**
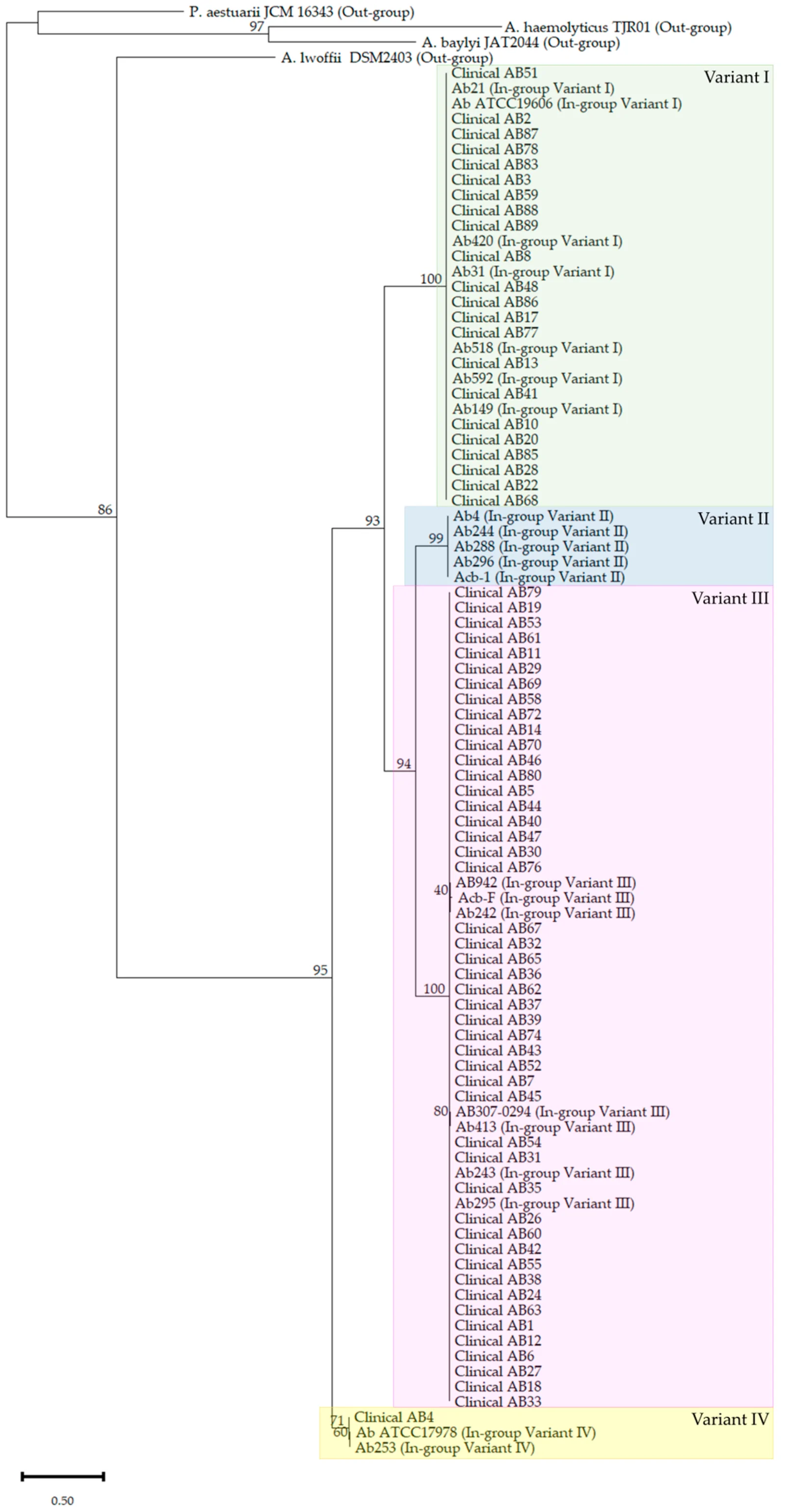
Phylogenetic tree showing the clonal relationships among clinical *A. baumannii* isolates and international reference strains. Numbers at internal nodes indicate bootstrap support values (%) based on 1000 replicates, reflecting statistical confidence. Variants I–IV are grouped in green, blue, pink, and yellow boxes, respectively. “In-group” denotes *A. baumannii* reference strains; “Out-group” denotes non-*A. baumannii* reference taxa used for rooting.

To elucidate the evolutionary relationships and clonal distribution of the clinical *A. baumannii* isolates, a phylogenetic analysis was conducted using 70 clinical isolates with available *carO* sequence data from the 77 confirmed *A. baumannii* isolates (designated Clinical AB1 through AB90, numbered non-consecutively). Phylogenetic reconstruction of the clinical *A. baumannii* isolates alongside international reference strains resolved four distinct phylogenetic groups (variants I to IV) (Figure 3), of which three (variants I, III, and IV) contained clinical isolates from this cohort. Non-*A. baumannii* out-group taxa (*Acinetobacter baylyi*, *Psychrobacter aestuarii* JCM 16343, and *Pseudomonas aeruginosa* PAO1), representing a range of genetic distances from the ingroup, were included to root the tree.

Variant I (green) formed the second-largest clade (22 isolates) including all 6 CSAB isolates, clustering with in-group reference strains AB19606, Ab21, Ab592, Ab149, Ab518, Ab31 ref, and Ab420 (100% support at the defining node) (Figure S1). Variant III (pink) was the largest clade, comprising 47 clinical isolates together with reference strains Ab295, Ab413, AB307-0294, Acb-F, AB942, Ab243, and Ab242 (100% bootstrap at the clade base) (Figure S2). Variant II (blue) contained only reference strains (Ab4 ref, Ab288, Acb-1, Ab296, and Ab244; 100% support), with no clinical isolates from this cohort. Variant IV (yellow; 100% bootstrap) comprised a single clinical isolate (AB4), grouping with reference strains Ab17978, and Ab253 as the most divergent phylogenetic group based on the *car*O sequence relative to the other in-group variants. Within Variant IV, the single clinical isolate (AB4) was phenotypically classified as CRAB. Notably, sequence alignment revealed near 100% identity between this resistant isolate and the known susceptible reference strains ATCC 17978 and Ab253 within this variant group, indicating that the extensive structural remodeling is inherent to this haplotype regardless of susceptibility status (Figure S3).

Demographic analysis initially showed no statistically significant difference in nationality distribution between the CRAB and CSAB groups (*p* = 0.2488; Table 2). To further investigate whether patient nationality was associated with distinct *carO* polymorphic profiles, a subgroup comparative analysis was performed. The analysis revealed no significant difference in *carO* variant distributions or mutation patterns between isolates recovered from Thai and Burmese patients (*p* = 0.2570), with both patient groups exhibiting a highly equivalent prevalence of the primary *carO* mutation clusters (Table S2).

### 2.6. AlphaFold2 Models of carO Variants Diverge Progressively from the 4RL9 Crystal Reference

**Figure 4:**
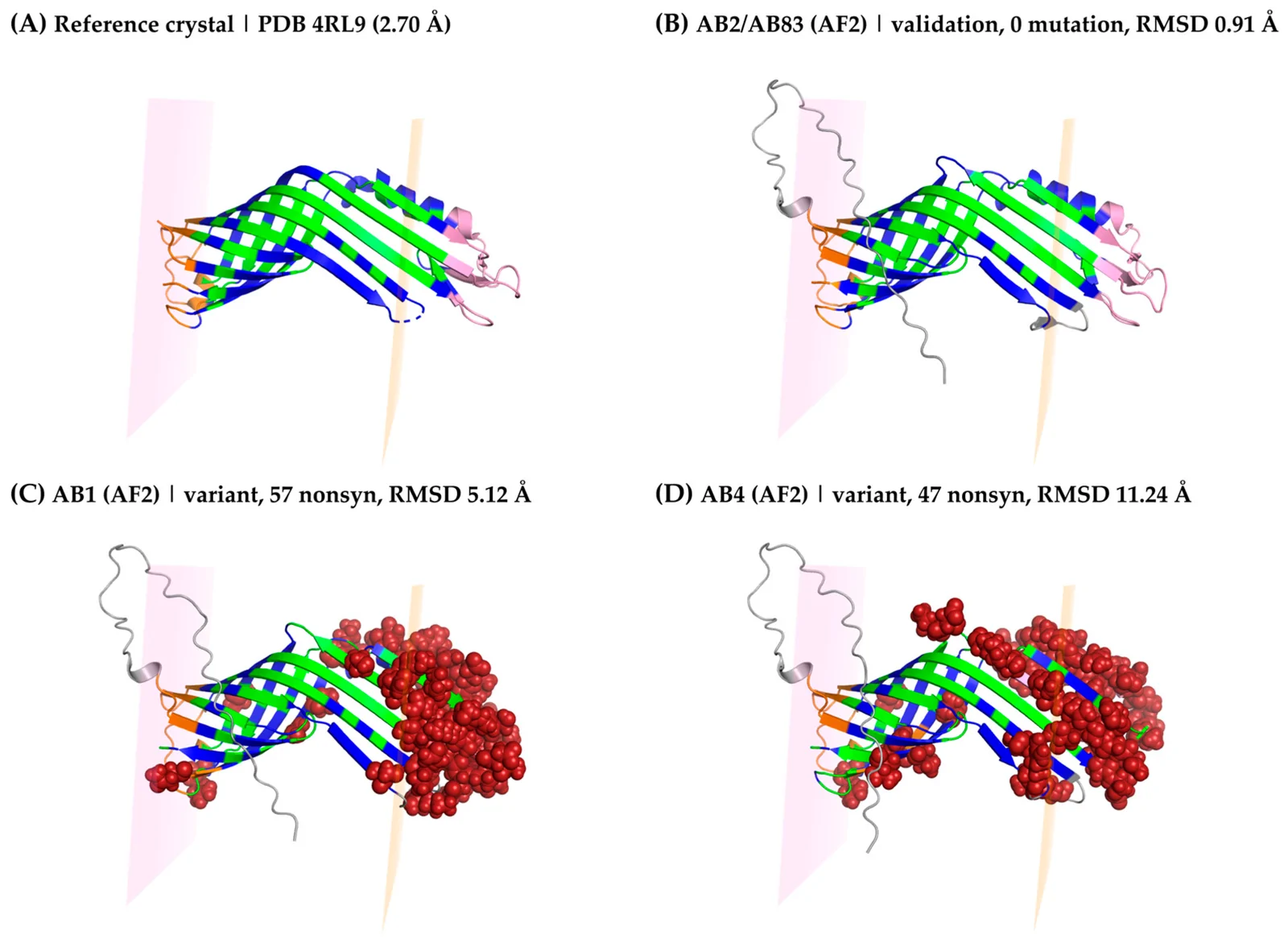
Structural consequences of *carO* haplotype variation modeled with AlphaFold2 against the 4RL9 crystal reference. PyMOL cartoon representations of CarO haplotypes rendered after global Cα superposition onto 4RL9 chain A. (**A**) *A. baumannii* CarO1 crystal structure (PDB 4RL9, 2.70 Å); (**B**) AlphaFold2 model of the CarO from AB2/AB83 isolates (validation; sequence identical to 4RL9); (**C**) AlphaFold2 model of the AB1 CarO isoform with 57 nonsynonymous positions relative to 4RL9, shown as firebrick spheres with the largest displacements localize to the C-terminal region; and (**D**) AlphaFold2 model of the AB4 CarO isoform, shown as firebrick spheres mark the 47 nonsynonymous positions across the extracellular loops, the C-terminal helix, and the barrel itself (maximum 23.12 Å at position 200). Cartoon coloring reflects membrane topology assigned from 4RL9: blue, lipid-facing β-strands; green, pore-facing β-strands; orange, extracellular loops and helices; pink, periplasmic segments; gray, unresolved or signal-peptide segments. Translucent orange (right) and pink (left) planes indicate the extracellular and periplasmic membrane boundaries derived from the barrel-axis analysis of 4RL9. Gray spheres, crystallographic detergent from 4RL9. Detergent, solvent, and heteroatoms from the deposited coordinates are omitted for clarity.

To assess whether the amino acid substitutions detected in *carO* alleles from *A. baumannii* isolates were associated with predicted structural changes, we predicted the tertiary structure of each haplotype with AlphaFold2 and superposed each model onto the *A. baumannii* CarO1 crystal structure (PDB 4RL9) in PyMOL. The AB2/AB83 haplotype was structurally indistinguishable from 4RL9 (Cα RMSD = 0.91 Å over 205 common residues; mean per-residue Cα distance = 0.78 Å), consistent with its identity to the reference sequence and confirming that the AF2 prediction pipeline reproduced the crystallographic fold (Figure 4A,B). The CarO isoform of the AB1 isolate showed moderate divergence from 4RL9 (RMSD = 5.12 Å, 202 residues; mean 4.08 Å, maximum 10.95 Å at position 213), with the largest Cα displacements concentrated in the C-terminal region and adjacent elements (Figure 4C). The CarO variant in the AB4 isolate was the most divergent haplotype in this study (RMSD = 11.24 Å, 198 residues; mean 8.54 Å, maximum 23.12 Å at position 200), with extensive rearrangement across the extracellular loops, the C-terminal helix, and the barrel itself (Figure 4D).

**Figure 5:**
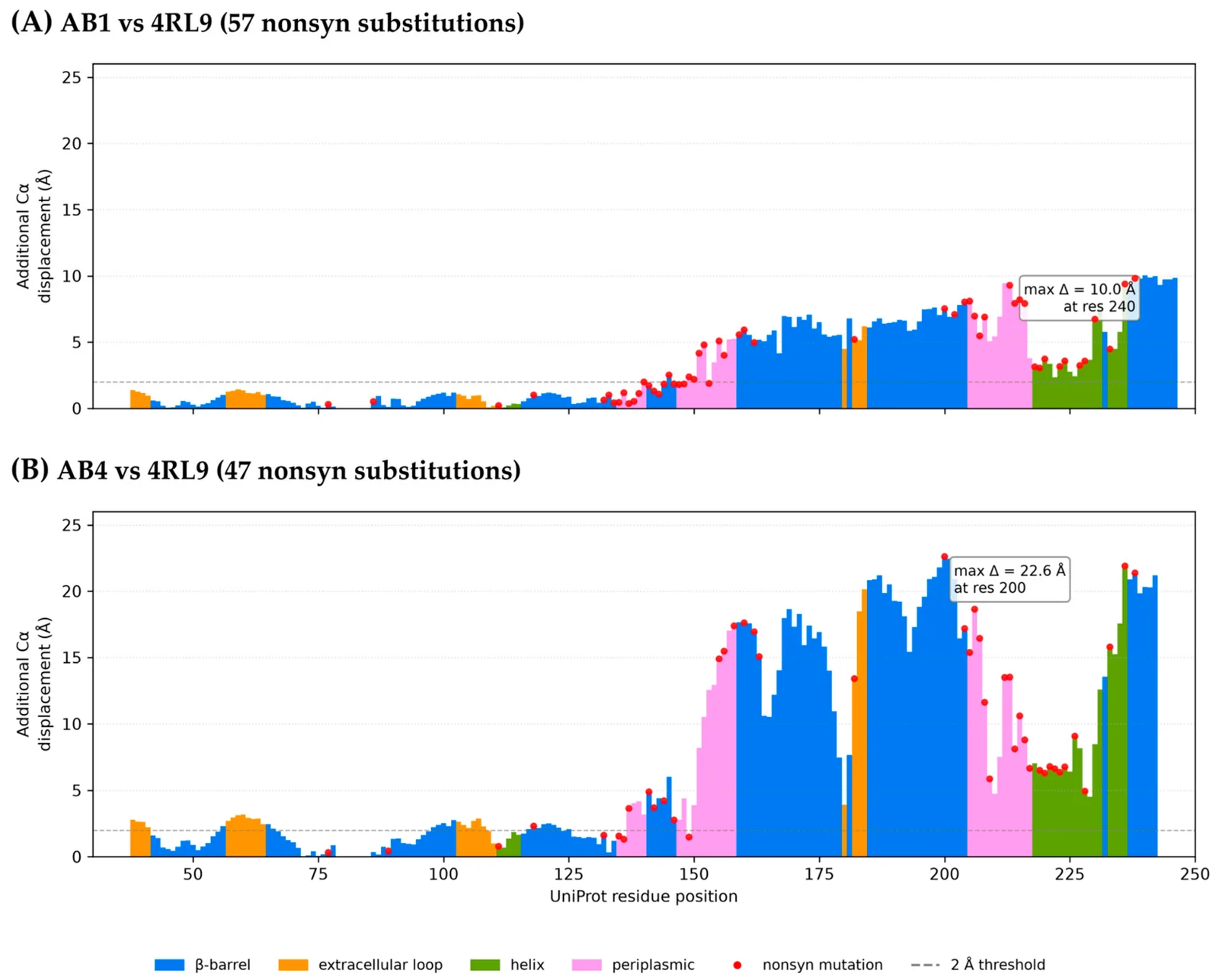
Structural change map in the 4RL9 reference frame—per-residue variant-specific Cα displacement. Per-residue Cα displacement of each variant AlphaFold2 model minus the displacement of the reference-matching AF2 model (AB2/AB83) at every position aligned to 4RL9, thereby isolating changes attributable to variant sequence rather than to AF2/crystal disagreement. (**A**) AB1 vs. 4RL9 (*n* = 57 nonsynonymous positions) and (**B**) AB4 vs. 4RL9 (*n* = 47). Bars are colored by 4RL9 secondary-structure region: β-barrel, blue; extracellular loop, orange; helix, green; periplasmic space, pink. Red dots mark nonsynonymous mutation sites within the modeled range; in-frame indels are not annotated. Gray dashed line, 2 Å significance threshold. The maximum additional displacement per panel is labeled with its residue position and value. Backbone remodeling in AB1 is concentrated at the C-terminal region and helix (positions ~210–240); in AB4 it spans the entire modeled range with the largest excursions in the barrel (residue 200) and adjacent extracellular loops (residues ~180–210).

Per-residue variant-specific Cα displacement (variant AB2/AB83 baseline) is mapped onto the reference sequence in the structural-change map (Figure 5). In the AB1 isolate, the change is concentrated at the C-terminal region (peak Δ = 10.05 Å at residue 240) (Figure 5A) with the C-terminal helix and periplasmic segments also displaced (max Δ = 9.38 Å at residue 236; max Δ = 9.45 Å at residue 212), while the β-barrel core shifts only modestly (mean 3.30 Å over 124 residues, median 1.84 Å). In AB4, the predicted structural differences span every region of the structure, with the largest additional displacements in the β-barrel (max Δ = 22.61 Å at residue 200), the extracellular loops (up to 20.14 Å at residue 184), and the C-terminal helix (max Δ = 21.90 Å at residue 236). Relative to 4RL9, AB1 contains 57 amino acid substitutions and three deletions (residues 157, 158, and 210; final protein length, 246 aa); AB4 contains 47 amino acid substitutions and seven deletions (residues 139, 150–154, 210; final protein 242 amino acids as shown in Figure 5B). Mean pLDDT scores of the AF2 models remained high across all three haplotypes (96.4, 97.2, and 96.4 for AB2/AB83, AB1, and AB4, respectively), and all residues with Cα displacement > 5 Å had pLDDT ≥ 88.5 (mean 97.7 for AB1 and 96.9 for AB4), indicating that the observed displacements reflect high-confidence structural predictions rather than model uncertainty (Table S3).

### 2.7. Specific Amino Acid Substitutions Defining CarO Variants in Clinical Isolates

To identify the specific amino acid changes contributing to the major mutation clusters (73.44% and 75.00%) observed in CRAB isolates (Table S1), we analyzed the substitution patterns corresponding to our structural models. These high-frequency mutations were completely absent in all six CSAB isolates, all of which belonged to variant I.

The major mutation clusters are primarily represented by the AB1 (variant III) haplotype, which is the most prevalent clone among the CRAB isolates. Within this variant, the 73.44% (*n* = 47) cluster corresponded to specific N-terminal substitutions, namely T77S, S81K, T85S, and K86T. On the other hand, the 75.00% (*n* = 48) cluster was driven by extensive modifications in both the central (positions 132–162) and C-terminal regions. This central region exhibited highly complex sequence variations, including specific substitutions and deletions such as G154 and Q155. Additionally, significant C-terminal substitutions associated with this group included K200T, E202K, T204V, and Y248F.

The AB4 (variant IV) haplotype, however, displayed a rare and distinct mutation profile found in only a single isolate (1.56%). Although it shares a few critical mutations with the major variant III cluster (e.g., P111R, I118V, and Y248F), AB4 possessed its own unique substitution signature. The unique mutations included T77R, V80L, V82I, L89V, and M163L, along with a highly divergent sequence in the C-terminal region.

## 3. Discussion

Carbapenem resistance in *Acinetobacter baumannii* is typically multifactorial rather than attributable to a single determinant [7,20,21]. Although OXA-type carbapenemases represent the principal mechanism of carbapenem resistance in most contemporary CRAB populations, accumulating evidence indicates that non-enzymatic mechanisms, including alterations of the CarO porin, can substantially modulate carbapenem susceptibility [15,20]. The relative importance of these mechanisms varies across epidemiological settings and genetic backgrounds [14]. This study provides a molecular characterization of *carO* sequence variation associated with carbapenem resistance in *A. baumannii* clinical isolates from Tak Province, Thailand, addressing a regional gap noted in national surveillance data [19]. No statistically significant differences in the evaluated baseline characteristics were detected between the CRAB and CSAB groups with respect to nationality, gender, ward of admission, or specimen type, although the groups differed in sample size. The high prevalence of CRAB observed in this cohort was consistent with high resistance rates reported in national surveillance data; however, the single-center design precludes determining whether this pattern represents a broader regional or national trend [22]. These findings reinforce the need for strengthened infection prevention and control practices, antimicrobial stewardship, and continued molecular epidemiological surveillance to inform alternative therapeutic strategies for CRAB.

Another finding of this study concerns colistin resistance among the *A. baumannii* isolates examined, accounting for 3.90% (3/77) of all isolates. This rate considerably exceeds the national colistin resistance rate of 1.4% reported by the NARST for the period 2021–2023 [22]. Such a discrepancy is concerning, given that colistin remains one of the few antimicrobial agents retaining effective activity against XDR *A. baumannii*. Acquisition of colistin resistance may result in a pandrug-resistant (PDR) phenotype. Treatment options for PDR strains are severely limited. In such cases, combination therapy guided by synergy testing may serve as a last-resort strategy. However, this approach carries an increased risk of adverse effects [23].

The strong statistical association was between CarO amino acid variation and the CRAB phenotype (*p* = 0.0002), including two mutation clusters present in 73.44% (47/64) and 75.00% (48/64) of CRAB isolates, respectively, but absent from all six CSAB isolates. This is consistent with the established role of CarO as a carbapenem-associated porin in *A. baumannii* [12,15]. Alterations of this CarO channel, including missense and frameshift mutations, are among the best-characterized mechanisms of carbapenem resistance in this species [13,15,24,25]. However, the presence of the CRAB isolates, e.g., AB4, with a *carO* sequence nearly identical to the known carbapenem-susceptible reference haplotype, (ATCC 17978 and Ab253) further supports the multifactorial nature of carbapenem resistance, suggesting that such isolates may rely on other mechanisms, such as carbapenemase production or efflux pump overexpression [10,11]. This mutational pattern was also reflected in *carO*-based phylogenetic clustering. The dominant variant (variant III) of the clinical isolates comprised most of the CRAB isolates and clustered with several international reference strains, including AB242 [6,14]. Interestingly, the variant I contained every CSAB isolate along with CSAB ATCC19606, suggesting that CarO porin in CSAB was highly conserved. These findings indicate that the CRAB burden here is driven by the dissemination of specific *carO* variants that are commonly reported in global carbapenem-resistant isolate.

The structural data offers a probable mechanistic bridge between these genotypic patterns and porin structure. Zahn et al. [12] showed by crystallography that CarO forms a closed, eight-stranded beta-barrel whose divergent residues concentrate in a large extracellular loop domain. The capacity of CarO to accommodate imipenem is governed by primary sequence rather than by a generic open channel [12]. In the present isolates, the AlphaFold2 models showed this architecture in the reference and AB2/AB83 haplotypes, which shared the same *carO* sequence as the reference (0% mutation). The presence of a CRAB isolate with a reference-like *carO* sequence and predicted structure further supports the contribution of other resistance mechanisms, such as carbapenemases or efflux pump overexpression [10,11]. For the clinical AB1 and AB4 isolates, AlphaFold2 models showed progressively larger C-α displacements concentrated in regions identified as functionally important: the extracellular loops, the C-terminal helix, and the barrel itself. These might affect the porin structure to reduce the accommodation of carbapenem [12]. The clinical AB4 was the sole representative of variant IV and exhibited the largest predicted structural rearrangements among the analyzed haplotypes.

Furthermore, our subgroup analysis revealed that *carO* polymorphic patterns and phylogenetic lineages were consistent between isolates recovered from Thai and Burmese patients (Table S2). In our view, this genetic similarity reflects a shared pool of CRAB strains within the border corridor between Thailand and Myanmar. Given the frequent population movement in the Mae Sot region, resistant pathogens can easily circulate across national boundaries regardless of patient nationality. This observation highlights a clear reality that antimicrobial resistance in border areas is a regional challenge for epidemiological surveillance. As a result, hospitals located near the border act not only as primary care facilities but also as key monitoring sites for regional resistance trends. Establishing joint surveillance networks and collaborative prevention strategies will be important to help control the regional spread of carbapenem-resistant strains.

Several limitations should be acknowledged. As we focused only on CarO porin structure, other mechanisms of carbapenem resistance were not characterized. Therefore, these mechanisms could not be excluded in this cohort. The cohort was drawn from a single regional hospital over a defined period and included a relatively a small sample size, especially CSAB samples. We consider the low proportion of CSAB (9.09%) to reflect our specific sampling context rather than the overall hospital epidemiology, as most isolates were collected from high-acuity settings (e.g., intensive care and internal medicine units). We hypothesize that the intense antibiotic selective pressure inherent to these wards drives a higher prevalence of resistant strains. Consequently, this limited CSAB sample size reduces our statistical power to definitively exclude the presence of low-frequency mutations in susceptible isolates. Thus, this CarO data limits generalizability beyond Tak Province. Structural modeling was restricted to four representative haplotypes (with only three variants found in clinical isolates) rather than the full variants. With AlphaFold2-based inference, the predicted C-α displacements describe plausible conformational consequences of sequence change rather than direct evidence of altered channel function [12]. Finally, we acknowledge the partial amplification limitations observed in the seven isolates (6 CRAB and 1 CSAB) that yielded only faint or weak bands during initial PCR. Consequently, these isolates may produce the yield reads too short for NGS analysis. This reduced amplification efficiency may indicate large *carO* deletions, insertion (e.g., ISAba elements), sequence disruptions or other sequence abnormalities impairing proper primer binding. These specific genetic anomalies were not resolved by long-read sequencing or Southern blotting in the current study. Determining the precise nature of these missing sequence coverages, whether they represent true gene deletions or sequencing artifacts, remains a critical step for future functional characterization to establish the exact association.

Taken together, phylogenetic clustering, mutation mapping, and structural modeling suggest that CarO sequence variation is associated with conformational differences in functionally important regions of the porin. However, these findings indicate an association rather than direct causation, and additional functional studies are required to determine the contribution of CarO variation to carbapenem resistance. Future work should prioritize functional validation of the highest-impact haplotypes identified here, using heterologous expression together with electrophysiological or liposome-based transport assays of the kind established by Zahn et al. [12]. Whole-genome sequencing or transcriptomic profiling with expression studies might be performed to resolve co-occurring carbapenemase and efflux determinants across the cohort [26,27] and to expand surveillance to additional provinces to determine whether the variant III-dominated clones reflect only localized transmission or a broader pattern.

## 4. Materials and Methods

### 4.1. Bacterial Isolates, Cultures and Antimicrobial Susceptibility Testing

Eighty-three clinical isolates of *A. calcoaceticus-baumannii* complex, identified by a VITEK^®^ 2 Compact automated system (bioMérieux, Lyon, France), were included in this study. Pure colonies were collected at the Clinical Microbiology Laboratory of Mae Sot Hospital, Tak, Thailand in August–December 2025. All bacteria were grown overnight on blood agar (Oxoid, Basingstoke, UK) at 35 ± 2 °C. For fresh culture, the bacteria were cultured in Mueller–Hinton broth (MHB; Oxoid, Basingstoke, UK) until logarithmic phases and then adjusted to achieve a 0.5 McFarland standard turbidity using a densitometer (Biosan, Riga, Latvia). *A. baumannii* ATCC 19606 was used as a control strain. All isolates kept in Cryoinstant tubes (Deltalab, Barcelona, Spain) were stored at −80 °C for further analysis.

Antimicrobial susceptibility testing was also performed using the VITEK^®^ 2 Compact automated system (bioMérieux, Lyon, France) according to the manufacturer’s protocols. A panel of seven antibiotics, including ceftazidime (CAZ), ceftriaxone (CRO), ciprofloxacin (CIP), imipenem (IPM), meropenem (MEM), doripenem (DOR), and colistin (CL), was tested. The CRAB group was defined as resistant to all three carbapenems (imipenem, meropenem, and doripenem) while the CSAB group was sensitive to those at the same time, as per the interpretation according to the Clinical and Laboratory Standards Institute guidelines [28]. The clinical isolates were also classified as non-resistant, MDR or XDR strains [29].

### 4.2. Genomic DNA Purification

Overnight cultures of each isolate in MHB were harvested at 12,000 g for 5 min. The genomic DNA (gDNA) was isolated using a Genomic DNA Isolation Kit (Bio-Helix, Taipei, Taiwan) according to the manufacturer’s instructions [30]. The gDNA concentrations were then analyzed using a Microvolume Spectrophotometer (Titertek-Berthold, Pforzheim, Germany) [26]. Only gDNA samples with good purity (A_260_/A_280_ ratios of around 1.9 ± 0.2) were kept at −20 °C for further analysis.

### 4.3. PCR Technique for Amplifying bla_OXA-51_-like and carO Cassettes

The *bla*_OXA-51_-like *and carO genes* were amplified in a LineGene 9600 Plus Real-Time PCR Detection System (Bioer Technology, Hangzhou, China) using primers shown in Table 3. All PCR reactions were performed using a SsoAdvanced Universal SYBR^®^ Green Supermix (BIO-RAD, Hercules, CA, USA). PCR reactions were mixed with 10 µL of the Master mix, 0.3 pmol/µL each of the forward and reverse primers, 1 µL of template DNA (corresponding to 30–200 ng of genomic DNA per reaction), and sterile DNase/RNase-free water up to a total volume of 20 µL. The PCR conditions were set in the Linegene 9600 Plus thermal cycler (Bioer Technology Co., Ltd., Zhejiang, China) as shown in Table 4. PCR products were loaded onto 1.5% agarose gels electrophoresis. The electrophoresis system was run at 110 V for 30 min, and the DNA bands were visualized using a gel electrophoresis imaging system (Aplegen, Ramsey, NJ, USA).

### 4.4. DNA Sequencing and Mutational Analysis

The PCR products of *carO* cassettes from all 77 clinical *A. baumannii* isolates were delivered to U2Bio (Seoul, Republic of Korea) for fast next-generation sequencing technology (Fast NGS). From FASTA raw sequences, only open reading frame (ORF) sequences of the *carO* gene in the 5′ to 3′ direction were retrieved using SnapGene software version 8.2.1 (GSL Biotech LLC, Chicago, IL, USA). Multiple sequence alignments (only 70 sequences) were performed in both nucleic acid and translated protein sequences using MAFFT and Clustal Omega algorithms, respectively. The aligned sequences were subsequently identified for mutations, compared to *carO* gene of *A. baumannii* ATCC19606 (accession no. CP046654.1).

### 4.5. Phylogenetic Analysis

The 70 trimmed sequences of *carO* were aligned against those of two ingroup sequences of *A. baumannii* and four outgroup sequences of other related microorganisms (accession numbers shown in Table 5), retrieved from GenBank. The phylogenetic tree was reconstructed using MEGA version 12.1 software (https://megasoftware.net/) with the Maximum Likelihood model. To assess the reliability of the tree, the bootstrap method was applied with 1000 replicates. Bootstrap values were calculated to provide confidence estimates for each branch in the tree.

### 4.6. CarO Sequence and Structural Analysis (4RL9 Reference)

The *carO* gene sequences of the four representative isolates (AB1, AB2, AB4, AB83) were translated with BioPython v1.87 and aligned to the *A. baumannii* CarO1 crystal reference PDB 4RL9 (UniProt A6XB80, 2.70 Å) using MAFFT v7.526 (L-INS-i). Per-sample amino acid substitutions and in-frame indels were cataloged relative to the reference and grouped into unique protein haplotypes. Secondary structure was assigned with DSSP (mkdssp v4.6.1) and a barrel-frame membrane topology was derived from the aromatic-belt residues (periplasmic belt Tyr87/Tyr148/Tyr206 at axial ≈ −19.4 Å; extracellular belt Trp57/Tyr117/Phe177/Trp185/Phe247/Tyr248/Trp249 at axial ≈ +24.9 Å; membrane span 44.3 Å) to classify each position as extracellular, membrane (lipid- or pore-facing), or periplasmic. Evolutionary tolerability of each nonsynonymous mutation was scored with the ESM-1v protein language model (esm1v_t33_650M_UR90S_1). Three-dimensional structures of unique haplotypes were predicted with AlphaFold2 (monomer preset, reduced databases; UniRef90, MGnify 2022-05, small BFD; PDB70/mmCIF template set) on high-performance compute infrastructure via the Biomni HPC gateway (biomni.tool.hpc_run_tool) and superposed onto the crystal reference in PyMOL v3.1.0 to compute per-residue Cα displacement and RMSD; per-residue pLDDT scores were extracted to assess prediction confidence. Variant-specific structural change was isolated by subtracting the reference-matching AF2 model’s per-residue displacement (AB2/AB83 baseline, which is 100% identical in sequence to 4RL9) from each variant, so that the residual reflects changes attributable to the variant sequence rather than to AF2/crystal disagreement. The full pipeline (Python 3.11, R 4.4.3) was implemented with AI-assisted coding on the Biomni platform (Phylo).

### 4.7. Statistical Analysis

All statistical analysis and figure creation were performed using GraphPad Prism version 11.0 software (Boston, MA, USA). Descriptive statistics were used to describe the general characteristics of the data as numbers (frequencies), percentages, and averages (with standard deviation). Mutation patterns were compared between CRAB and CSAB isolates using Fisher’s exact test (reporting as *p*-value). To account for multiple testing, *p*-values were adjusted using the false discovery rate (FDR) procedure of Benjamini and Hochberg, reported as a *q*-value. Statistical significance was set at a *p*-value or *q*-value < 0.05.

## 5. Conclusions

In conclusion, this study successfully characterized the mutation patterns of the *carO* gene in *Acinetobacter baumannii* clinical isolates from Mae Sot Hospital, Tak Province, Thailand. The predominant CarO mutation type in CRAB was missense, revealing a statistically significant association (*p* = 0.0002) between distinct polymorphic variations and the carbapenem-resistant (CRAB) phenotype. Phylogenetic reconstruction resolved three distinct clinical lineages with variant III predominating, indicating the widespread dissemination of specific *carO* variants (e.g., variants I and III) that closely resemble those globally reported in carbapenem-resistant strains. Furthermore, computational structural modeling using AlphaFold2 predicted progressive structural divergence and substantial remodeling of the extracellular loops, C-terminal helix, and β-barrel channel in resistant variants relative to the PDB 4RL9 susceptible reference structure. These findings provide critical regional molecular epidemiological data on CarO porin-mediated carbapenem resistance in Thailand. However, functional validation of these structural variants is needed to optimize future surveillance and alternative therapeutic strategies.

## Figures and Tables

**Table 1:** Clinical information of patients infected with *A. calcoaceticus-baumannii* complex isolates at the time of first identification.

Parameters (Total = 83 Isolates)		Frequency (*n*, %)
Nationality	Thai	52 (62.65)
	Burmese	31 (37.35)
Gender	Male	55 (66.27)
	Female	28 (33.73)
Age (mean ± standard deviation)		57.51 (20.68)
Ward	Internal medicine wards	37 (44.58)
	Intensive care unit	29 (34.94)
	Surgical wards	12 (14.46)
	Other wards	5 (6.02)
Specimen type	Sputum	54 (65.06)
	Pus	17 (20.48)
	Blood	4 (4.82)
	Body fluid	4 (4.82)
	Urine	4 (4.82)

**Table 2:** General information on both CRAB and CSAB groups isolated from the patients.

Parameters		Frequency (*n*, %)			*p*-Value
		Total *n* = 77	CRAB *n* = 70	CSAB *n* = 7	
Nationality	Thai	49 (63.64)	46 (65.71)	3 (42.86)	0.2488
	Burmese	28 (36.36)	24 (34.29)	4 (57.14)	
Gender	Male	54 (70.13)	50 (71.43)	4 (57.14)	0.4203
	Female	23 (29.87)	20 (28.57)	3 (42.86)	
Ward	Internal medicine wards	33 (42.86)	29 (41.43)	4 (57.14)	0.4536 (Internal Medicine vs. others)
	Intensive care unit	28 (36.36)	26 (37.14)	2 (28.57)	
	Surgical wards	11 (14.29)	10 (14.29)	1 (14.29)	
	Other wards	5 (6.49)	5 (7.14)	0 (0.00)	
Specimen type	Sputum	51 (66.23)	46 (65.71)	5 (71.43)	1.0000 (Sputum vs. other types)
	Pus	16 (20.78)	14 (20.00)	2 (28.57)	
	Blood	3 (3.90)	3 (4.29)	0 (0.00)	
	Body fluid	3 (3.90)	3 (4.29)	0 (0.00)	
	Urine	4 (5.19)	4 (5.71)	0 (0.00)	

**Table 3:** Oligonucleotide primers used in this study.

Gene of Interest	Primer Name	Oligonucleotide Sequences (5′ to 3′ Direction)	Expected Size (bp)	References
*carO*	carO_Seq_F carO_Seq_R	GCTGAGACATGATCTTCCTTCC AAACGAGCCGAAGCTCG	1086	This study
*bla*_OXA-51_-like	blaOXA51_F blaOXA51_R	TAATGCTTTGATCGGCCTTG TGGATTGCACTTCATCTTGG	353	[31]

Note: F, forward primer; R, reverse primer. The broad compatibility of *carO* primers was in silico validated against a global database using a nucleotide BLAST (BLASTn) analysis (https://blast.ncbi.nlm.nih.gov/Blast.cgi, accessed on 22 July 2026) (Table S4).

**Table 4:** PCR conditions used in this study.

Gene	Purpose	Initial Denaturation	Conditions for Thermal Cycles				Final Extension
			Cycles	Denature	Anneal	Extend	
*carO*	Sequencing of *carO* cassette	94 °C for 3 min	35	94 °C for 20 s	55 °C for 25 s	72 °C for 1 min 15 s	72 °C for 3 min.
*bla*_OXA51_-like	For genotypic identification as *A. baumannii*	98 °C for 2 min 20 s	40	98 °C for 20 s	51 °C for 25 s	72 °C for 25 s	

**Table 5:** Various strains of *carO* genes used for the phylogenetic tree in this study.

Strains	Accession No.	Variant	Phenotype	
			IMP	MER
*A. baumannii* ATCC19606	CP046654.1	I	S	S
*A. baumannii* Ab244	AY684798	II	S	S
*A. baumannii* Ab242	DQ309876	III	R	R
*A. baumannii* ATCC17978	CP053098.1	IV	S	S
*A. baumannii* Ab21	DQ309875	I	R	S
*A. baumannii* Ab518	FJ652389	I	R	R
*A. baumannii* Ab592	FJ652385	I	S	S
*A. baumannii* Ab31	FJ652386	I	I	S
*A. baumannii* Ab149	FJ652387	I	S	S
*A. baumannii* Ab420	FJ652388	I	R	R
*A. baumannii* Ab4	FJ652392	II	R	I
*A. baumannii* Ab288	FJ652391	II	S	S
*A. baumannii* Ab296	FJ652390	II	S	S
*A. baumannii* Acb-1	DQ642020	II	R	I
*A. baumannii* Ab243	FJ652394	III	R	R
*A. baumannii* Ab295	FJ652393	III	S	S
*A. baumannii* Ab413	FJ652395	III	R	R
*A. baumannii* Acb-F	DQ642021	III	S	S
*A. baumannii* AB942	EF646275	III	S	S
*A. baumannii* AB307-0294	CP001172	III	S	S
*A. baumannii* Ab253	EF537047	IV	S	S
*Acinetobacter lwoffii* DSM2403	NZ_CP118963.1	Outgroup	ND	ND
*Acinetobacter baylyi* JAT2044	CP104734.1	Outgroup	ND	ND
*Acinetobacter haemolyticus* TJR01	CP038009.1	Outgroup	ND	ND
*Psychrobacter aestuarii* JCM 16343	BAAAFR010000005.1	Outgroup	ND	ND

Note: S, susceptible; I, intermediate; R, resistant; ND, not determined.

## Data Availability

The data supporting the current study are available from the corresponding author (J.J.), upon reasonable request.

## Supplementary Materials

The following supporting information can be downloaded at: https://www.mdpi.com/article/10.3390/antibiotics15090828/s1, Figure S1: Multiple alignment of CarO sequences from clinical isolates in Variant I, compared to *A. baumannii* ATCC19606; Figure S2: Multiple alignment of CarO sequences from clinical isolates in Variant III, compared to *A. baumannii* ATCC19606; Figure S3: Multiple alignment of CarO sequences from clinical isolates in Variant IV, compared to *A. baumannii* ATCC19606; Table S1: Specific mutation patterns of CarO amino acids in clinical isolates; Table S2: Subgroup analysis of nationality and mutation patterns; Table S3: per residue pLDDT 4RL9; Table S4: BLASTn for in silico primer validation.
