# Supplementary material for: Molecular and Structural Insights of carO Gene Variations in Carbapenem-Resistant Acinetobacter baumannii
Source: Antibiotics (Basel). 2026 Aug 26;15(9):828. doi: 10.3390/antibiotics15090828 (PMC13603979; doi:10.3390/antibiotics15090828)

Figure S1. Multiple alignment of CarO sequences from clinical isolates in Variant I, compared to *A. baumannii* ATCC19606

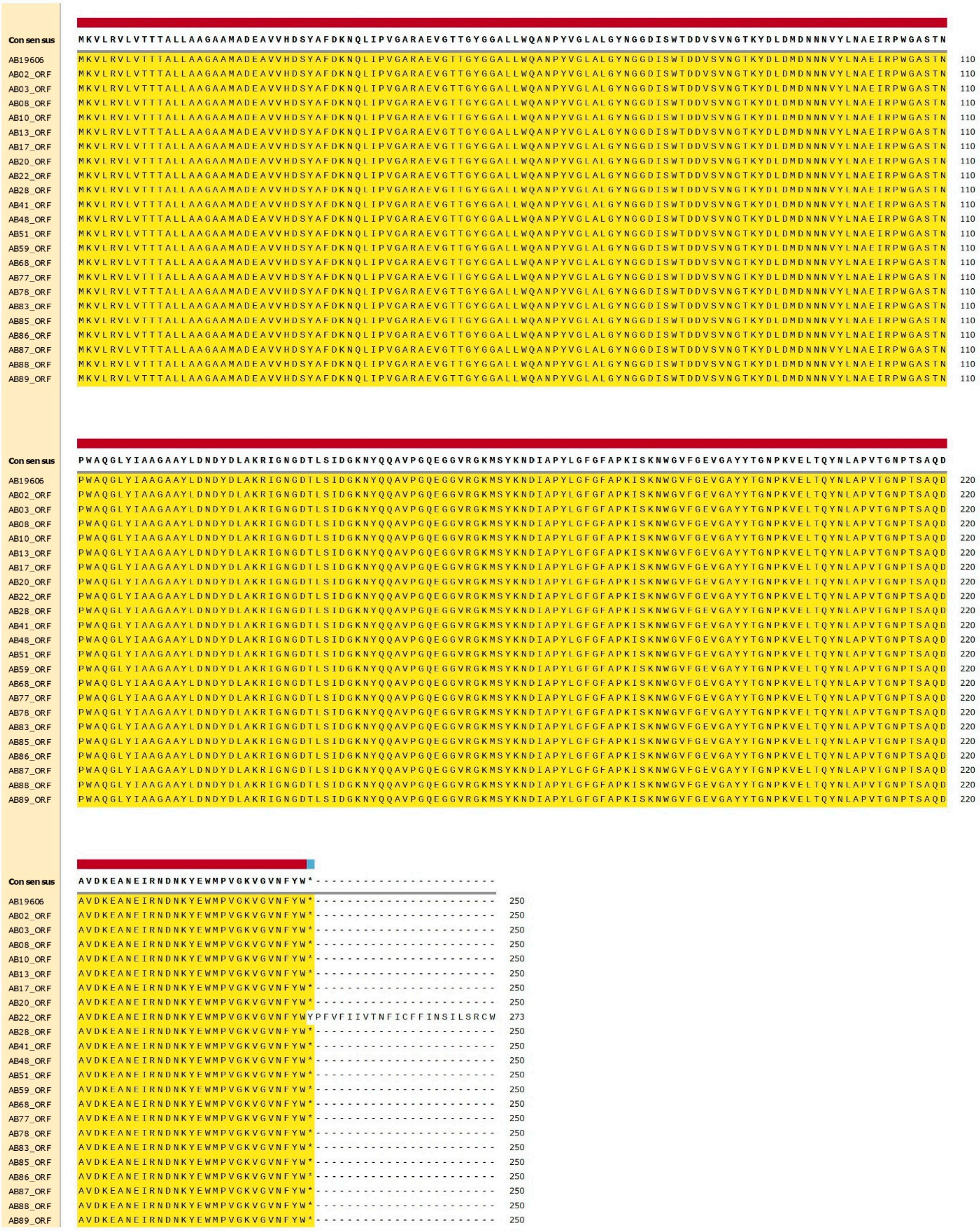

Figure S2. Multiple alignment of CarO sequences from clinical isolates in Variant III, compared to *A. baumannii* ATCC19606

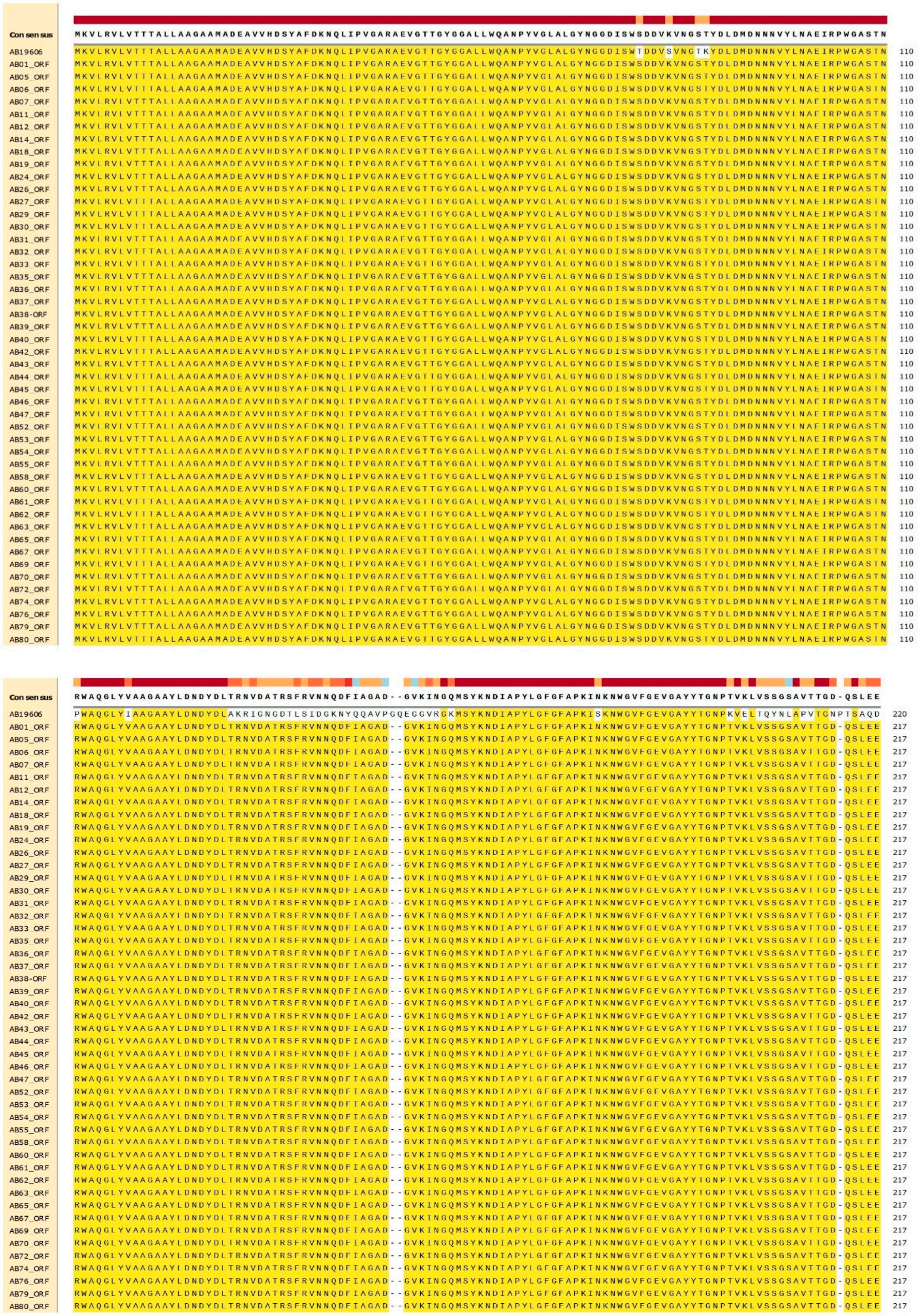

Figure S2 (Continue). Multiple alignment of CarO sequences from clinical isolates in Variant III, compared to *A. baumannii* ATCC19606

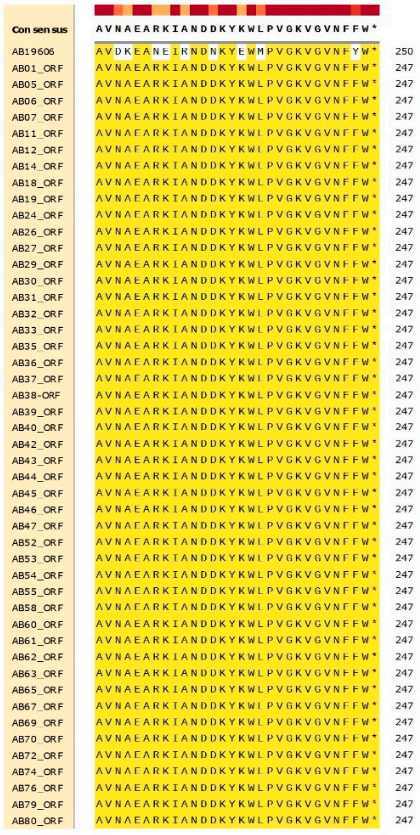

Figure S3. Multiple alignment of CarO sequences from clinical isolates in Variant IV, compared to *A. baumannii* ATCC19606

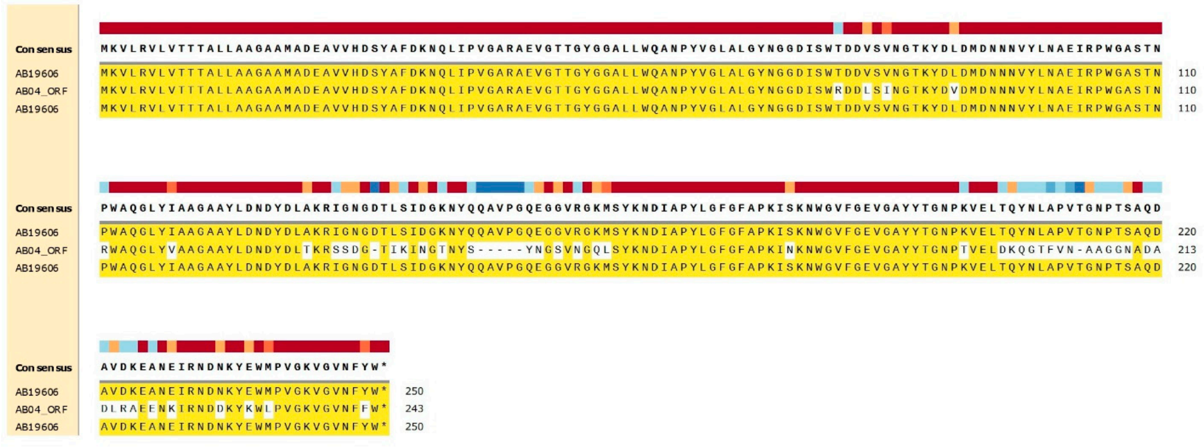

Supplement: Supplementary file 1 [file antibiotics-15-00828-s001.zip › Figure S1-S3 Multiple alignment of CarO .pdf]
